# Surgery of temporomandibular joint under local anaesthesia

**DOI:** 10.4103/0970-0358.44941

**Published:** 2008

**Authors:** Kalpesh J. Gajiwala

**Affiliations:** Holy Family Hospital, Bandra; Ramkrishna Mission Hospital, Khar; Saifee Hospital, Charni Road; AYJNIHH, Bandra, Mumbai, India

**Keywords:** Local anaesthesia, temporomandibular joint ankylosis, temporomandibular joint arthroplasty

## Abstract

Temporomandibular joint ankylosis is a debilitating disorder arising from an inability to open the mouth. This leads to poor nutrition, poor dental hygiene, and stunted growth. Anaesthesia, especially general anaesthesia, is very difficult to administer. There is a lack of direct visualization of the vocal cords, tongue fall following relaxation, and an already narrowed passage due to a small mandible, which makes even the blind nasal intubation difficult. There are various techniques described in literature to overcome these challenges, failing which, one needs to do tracheostomy. All the risks of difficult intubation and general anaesthesia can be avoided if the surgery is done under local anaesthesia. A simple but effective method of successful local anaesthesia is described, which allows successful temporomandibular joint reconstruction.

## INTRODUCTION

Temporomandibular joint ankylosis requires surgery to restore mouth opening. Inability to open the mouth results in an inability to maintain dental hygiene and to chew solid food. This leads to dental caries, malocclusion, weight loss, and stunted growth. The problems in temporomandibular joint ankylosis surgery are general anaesthesia and the requirement for nasal intubation. In the absence of visualisation of the vocal cords, it is difficult to intubate,[1–3] and often, tracheostomy is required.[4] Temporomandibular joint arthroplasty can be done under local anaesthesia, thus avoiding the need for difficult, often blind, nasal intubation and associated trauma to the pharynx and vocal chords, and possible emergency tracheostomy wherever the facility for fibre optic endoscope is not available. Local anaesthesia is simple, safe, and effective.

## MATERIALS AND METHODS

From June 1987 to April 2007, 21 cases (aged 4–30 years), of which most were within 10–16 years age group, of temporomandibular joint surgery were done under local anaesthesia. Nineteen cases were of temporomandibular joint bony ankylosis, one case was of bilateral recurrent dislocation, and the remaining one of unilateral recurrent dislocation. Sixteen cases of unilateral and three cases of bilateral temporomandibular joint ankylosis were treated. In most cases, the initial inter incisor distance was ≤ 4 mm and the duration of involvement ranged from 4–13 years, an average of 6 years. The most common cause was trauma in eight cases, followed by infection in four, but the aetiology was unknown in the rest. Most of these patients belonged to poor socioeconomic groups, and were malnourished and small for their age.

## TECHNIQUE

For infiltration, a solution was made up of mixture of xylocaine 2% with 1:200,000 adrenaline 7cc plus 0.5% bupivacaine 3 cc, and hyaluronidase 1500 units dissolved with 1 cc of normal saline. First, the preauricular area was infiltrated along the line of incision with about 1.5 ml. of the above solution. Then, the needle was inserted just in front of the lower end of the tragus and passed horizontally below the zygomatic arch. The soft tissues were infiltrated with about 3–4 ml. of the solution and about 1.5–2 ml. were infiltrated into the subcutaneous plane over the zygomatic arch as this was stretched during retraction. After palpating the notch, the needle was then reinserted in front of the condyle to a depth of about 4 cm where the mandibular nerve is located, and about 3–5 ml of the infiltration solution were injected taking care to avoid intravascular injection. This injection can also be given with the above solution without adrenaline. About 1 ml. was infiltrated around the neck of the mandible while withdrawing the needle. The slow infiltration with a long 24 or 26 G needle and its insertion through a previously anesthetised zone kept the patient comfortable and cooperative. No sedation was required or given and it was frequently not necessary to attempt a mandibular nerve block. The needle could simply be passed in front of and behind the condyle and infiltrate the soft tissues around the ankylosed condyle.

**Figure 1:**
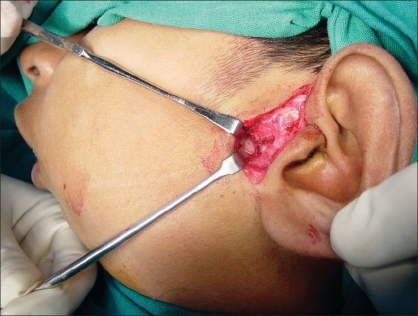
Preauricular incision

**Figure 2:**
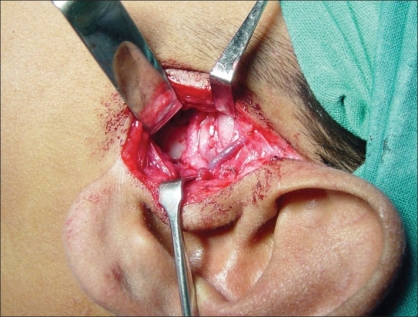
Isolation of auriculotemporal neurovascular bundle, keeping them posterior to the zone of dissection

**Figure 3:**
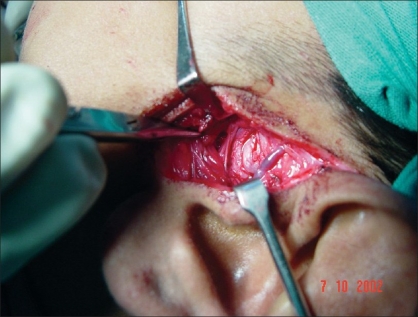
Exposure of ankylosed TMJ

**Figure 4:**
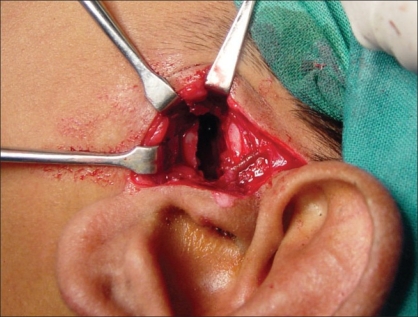
Creation of gap of around 5–7 mm between the condyle and new fossa

**Figure 5:**
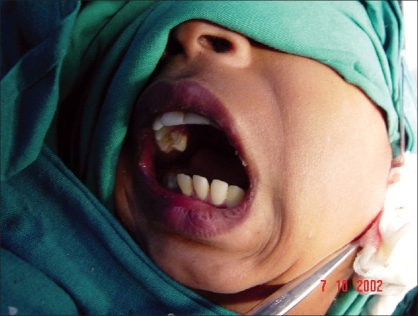
Patient is asked to open the mouth to verify completion of disjunction; note the creation of nasolabial fold indicating active facial nerve and muscles

**Figure 6:**
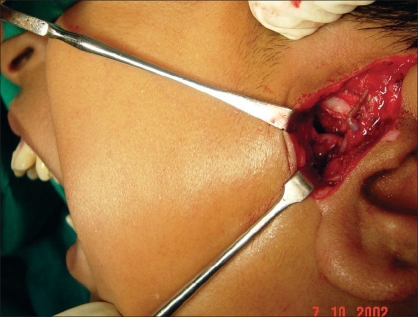
Lateral view to show the mouth opening and completion of disjunction

**Figure 7:**
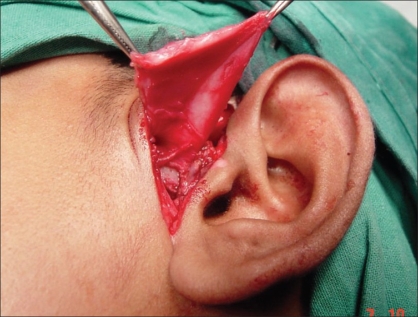
Elevation of inferiorly based temporalis facia flap

**Figure 8:**
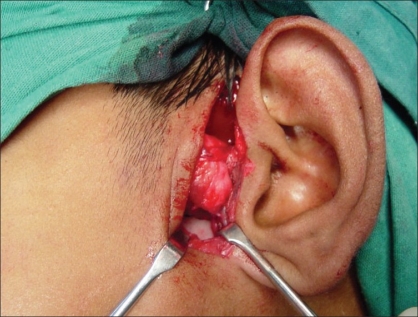
Insertion and fixation of temporalis facia into the new TMJ space

Preauricular incision was taken from the helical rim superiorly to the point where the ear lobe joins the face. [Figure 1] The incision was deepened and the superficial auriculotemporal vessels and nerve were identified and pushed posteriorly. A blunt dissection was carried out just below the zygomatic process in front of the tragus [Figure 2]. It was deepened until the condylar bone could be felt. In some cases, this dissection could be quite deep before one reached the condyle. Keeping the bone in sight with the help of small right-angled retractors, the window was widened in the forward direction, taking care to see and identify any branch of the facial nerve. If seen, these were gently dissected out of harm's way beneath the retractors. At this point, movement of the frontalis and orbicularis oculi could be checked—if they were active and their action was equal to their contralateral counterpart, the anaesthetised zone was considered not to have the facial branch that was likely to be the first affected. If any of the facial muscle showed paralysis or weakness, infiltration was considered to be extensive and an obvious advantage was lost. The soft tissues were dissected away from the condyle and the fused joint was exposed as much as possible [Figure 3]. With the help of a fissure burr, the condyle was cut and a 5–7 mm gap created [Figure 4]. If local anaesthesia was needed at any point of time during this procedure, it could be supplemented. Infiltration was considered if the mandibular block was either not given or not fully effective when one reached the medial aspect of the condyle. In this case, the injection needle was gently bent near the tip at right angles and inserted from behind the posterior border of the exposed condyle to carry out the infiltration of the medial aspect. In the same way, infiltration could also be done from the front of the condyle, which makes medial disjunction painless. After the gap was created, the patient was asked to open their mouth to confirm that disjunction was complete [Figures 5 and 6]. All sharp bone projections were evened out and a flap of temporalis fascia of adequate dimensions based inferiorly near the zygomatic arch was raised [Figure 7]. This flap was interposed between the condyle and the articular fossa [Figure 8]. The edges of the flap were sutured to the soft tissues in the front of and at the back of the newly created joint, so as to prevent the slippage of the interposed fascia out of the joint. The rest of the soft tissues were sutured over the turned-down fascial flap and the wound was closed. No coronoidectomy was done unless it too was found to be ankylosed. In this series, only one case, which had been previously operated with a costochondral graft, needed the coronoidectomy. Most often, the mouth opening achieved was around 12–18 mm of interincisor distance as no further soft tissue release was done [Figure 10]. A drain was usually not needed and an elastic crepe bandage was tied after the dressing.

The patient was postoperatively given antiinflammatory and analgesic agents, local icepacks, and asked to chew chewing gum. He was then asked to use a clothesline clip[5] as a jaw stretcher, which was to be reversed and put at the level of the molars. Later, a specially designed dynamic jaw exerciser was given.

## RESULTS

**Figure 9:**
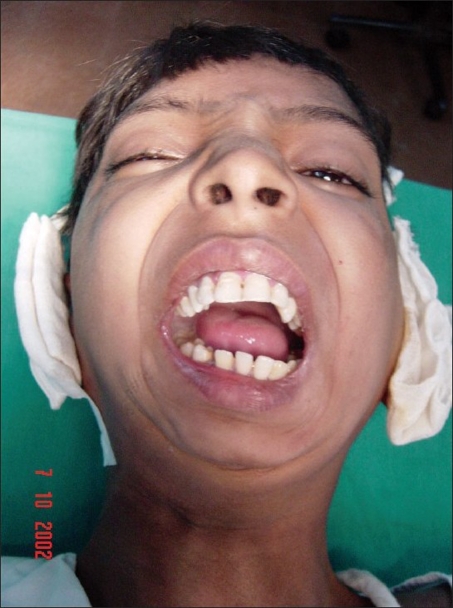
Mouth opening immediately postop, a case of bilateral TMJ ankylosis

The tissues were gradually stretched over 8–10 weeks with the dynamic jaw stretcher to obtain a mouth opening of 35–45 mm (average = 38 mm). Patients were followed up for six months to three years, with an average of nine months [Figures 9 and 11].

**Figure 10:**
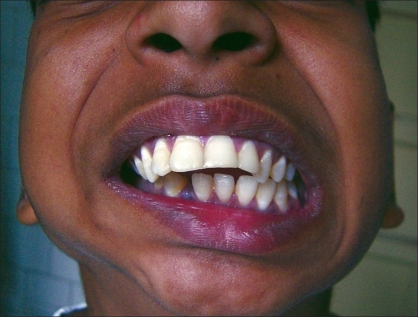
Preoperative view of the same patient

**Figure 11:**
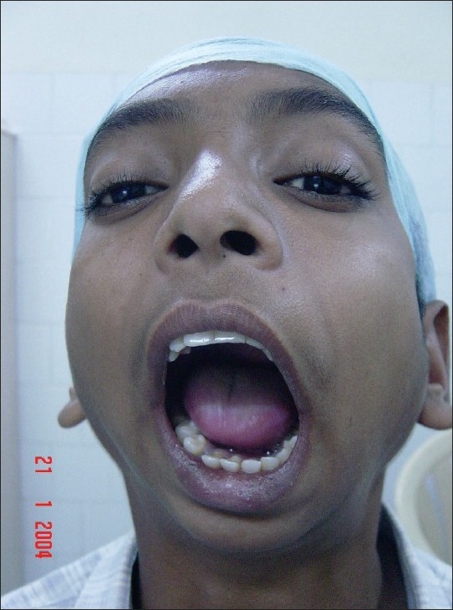
Postoperative view after about three months

## COMPLICATIONS

**Figure 12:**
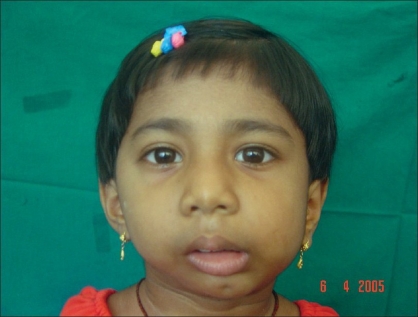
A four year-old girl weighing 11 kg, with left TMJ ankylosis

**Figure 13:**
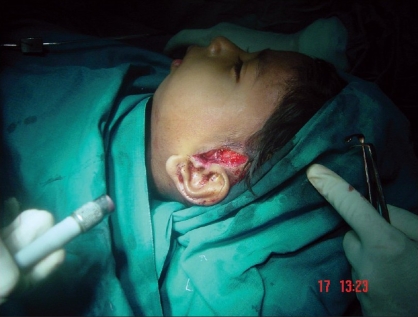
A promise of a chewing gum, soothing talk, and confidence-building by the surgeon and the anesthetist (Dr. Seema Panjabi); a gentle infiltration of anesthetic allowed the surgery to proceed

**Figure 14:**
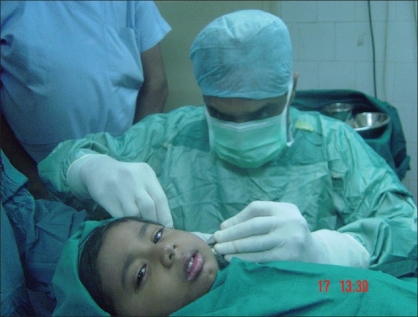
Frontal view of the same patient during surgery; note the absence of any intubation and awake look

**Figure 15:**
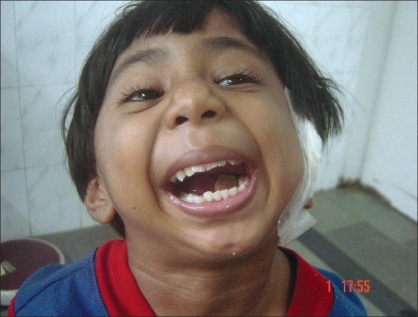
Ten days postop: happiness after a modest mouth opening and ability to chew foods and chewing gum

Two patients complained of partial loss of mouth opening during the morning hours and were asked to use the dynamic jaw exerciser regularly. The surgery was postponed in one adult patient due to oozing from the roof, for fear of accidentally entering the cranial vault. The dissected area was packed with gel foam and re-explored after two weeks without further problem. A four year-old child [Figures 12–15], had a relapse. She could not be provided adequate after care, as the mother could not look after the child due to a personal calamity in the family. The child also had a postoperative haematoma and infection. None of these patients had facial nerve injury or parotid gland injury or injury of the deeper vessels or nerves (except one). The patient with a failed costochondral graft, who had a diffusely fused condyle and coronoid process, (there was no sigmoid notch), had a loss of sensation in the right lower lip but showed partial recovery over eight months.

## DISCUSSION

Surgery of temporomandibular joint ankylosis under general anaesthesia poses a great challenge to anaesthesiologists. This surgery falls in the category of difficult intubation[1,2] as direct vocal cord visualisation is difficult due to an inability to open the mouth. There is associated retrognathia with a relatively large tongue, and a pseudomacroglossia in a confined space that narrows the pharyngeal passage.[6] Many patients suffer from obstructive sleep apnoea.[3,7] All of these factors make intubation much more difficult. Options are few and difficult: a blind nasal intubation,[8] fibre optic laryngoscope-assisted intubation,[1,9] bi nasopharyngeal airway[10,11] fluoroscope-aided retrograde placement of guide wire for tracheal intubation,[12] retrograde endotracheal intubation using a pharyngeal loop,[13] semi blind technique of nasal intubation,[14] and tracheostomy.[4] Blind nasal intubation has high failure rates[10,14,15] and chances of trauma to the air passage and larynx are high. Fibre optic laryngoscope is the best option but requires a set of costly endoscopes of various sizes; oxygenation between induction and intubation may be a problem.[1,9,14] and that too could fail.[16] Retrograde transtracheal puncture and guide wire, described first by Waters, can be difficult.[12,13,17] and has its own complications of tracheal bleed, which could be occasionally fatal.[18,19] Associated obstructive sleep apnoea is another challenge one has to deal with both during the intubation and during postoperative recovery.[3,7] Vas and Sawant[14] reported that 6/15 patients could not maintain their airway in a supine position and had to be turned into a lateral position for better ventilation. Duration of induction to intubation time can be quite high, causing anxiety to both the surgical and anaesthesia team. Many failed intubations may end up with tracheostomy.[4,17,20] In a multicentric Nigerian study, Ugboko *et al.* reported a 30.4% tracheostomy conversion rate.[4]

In light of all these factors, it could be a boon to both the medical team and the patient if temporomandibular joint ankylosis surgery could be done under local anaesthesia. Inferiorly based temporalis fascia or muscle-fascia interposition arthroplasty is a well accepted method and has been used primarily.[21–23] or following a failed implant or surgeries.[24] A costochondral graft has its own place in the armamentarium, but has been associated with high recurrence *vs* fascia, and has needed more repeat surgeries.[25] Its growth potential is unpredictable.[25] Whereas inferiorly based temporalis fascia or muscle-fascia flap can be easily obtained under the same local anaesthesia. Other forms of interposition materials[26–32] could also be safely used under local anaesthesia. Simple gap arthroplasty too can easily be done under local anaesthesia.[33]

**Figure 16:**
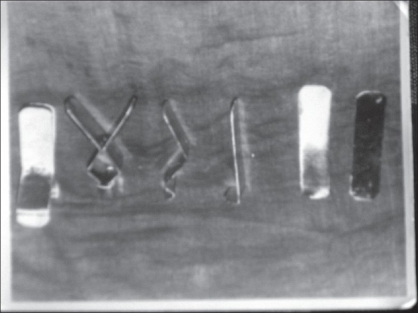
A jaw stretcher made of two flat aluminium strips bent in a lazy ‘z’, joined by a hinge joint. The outer surface was lined with a soft material to prevent damage to teeth. This was devised by the author in 1987

**Figure 17:**
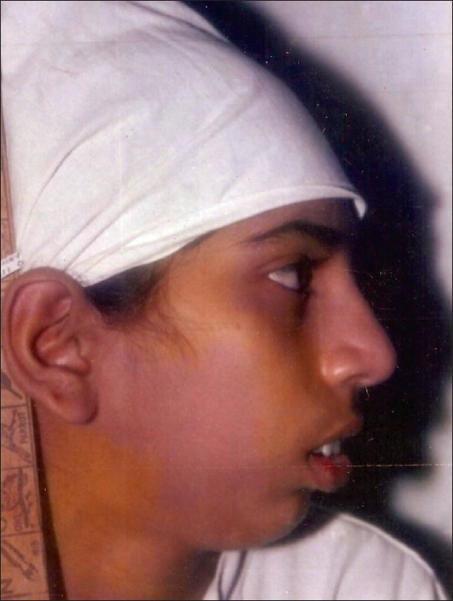
A case of TMJ ankylosis, right lateral view

**Figure 18:**
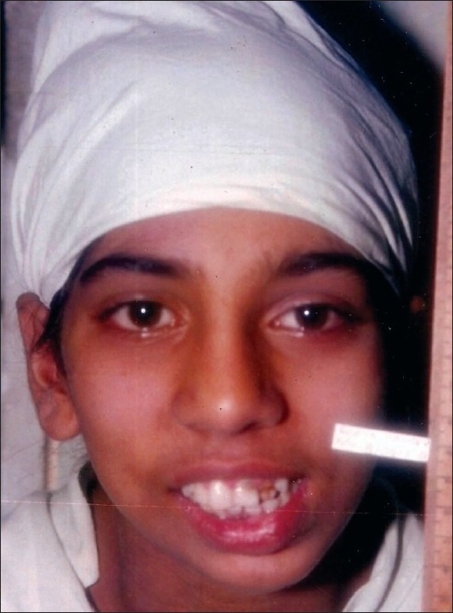
Same patient in frontal view: there is no interincisor clearance

**Figure 19:**
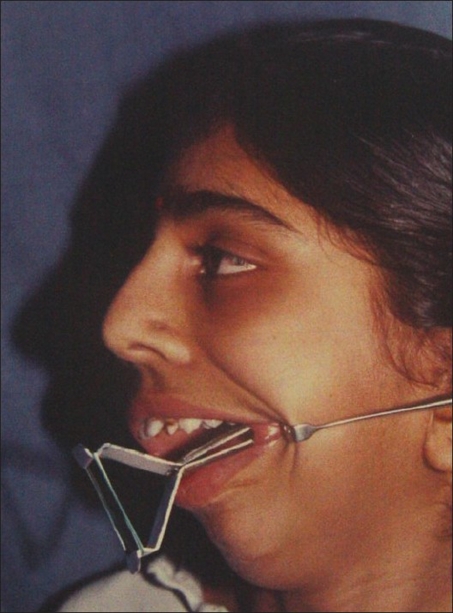
Using the jaw stretcher to improve mouth opening. Note the mouth opening in early post operative period

**Figure 20:**
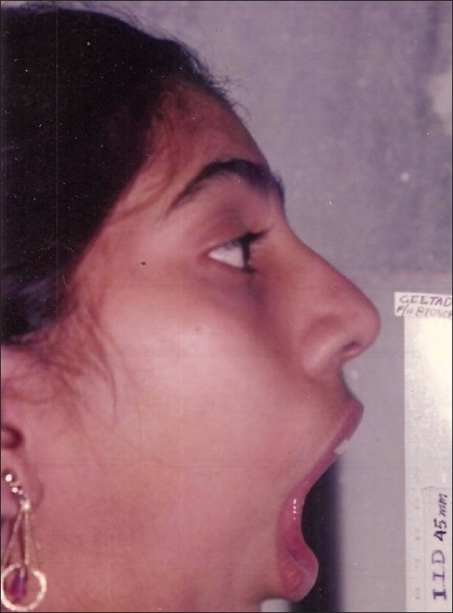
Final Mouth opening of about 45 mm inter-incisor distance, lateral view

**Figure 21:**
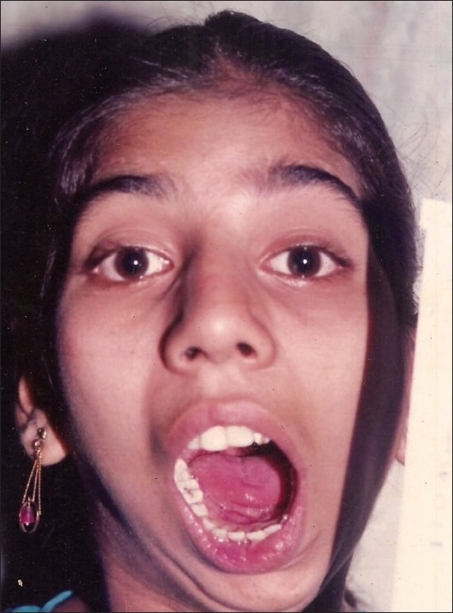
The same patient in front view. note the wide mouth opning

Anatomically there are very few nerves that need to be blocked: the auriculotemporal and the greater auricular nerve supply the skin and the soft tissue around the temporomandibular joint. The joint itself is supplied by the auriculotemporal nerve and some fibres from the masseteric branch of the mandibular nerve[34]. All these nerves can be blocked easily. The amount of local anaesthesia required is limited and can be given in incremental doses, and is rarely ever required at more than the maximally recommended dose. It is therefore very safe, simple, and reliable. Patient cooperation can be obtained with preoperative interaction and counselling. If anaesthesia is started with a finer needle (26 G) on an insulin syringe and given very slowly, even stretch pain of the injection can be eliminated, and further anaesthesia can similarly be extended through the previously anaesthetised zone. Slower injection results in a longer time period, ensuring enough time for vasoconstriction to happen, leading to an almost clear, bloodless field and is time-saving in the long run. The benchmark for doing surgery under local anaesthesia near the ear is already established by ENT surgeons who routinely do mastoid surgery under local anaesthesia. Burring of a bone seems to be a painless affair and is well tolerated by patients. Surgery under local anaesthesia is considered safer than general anaesthesia. In this series, coronoidectomy was not done and gradual soft tissue stretching achieved remarkable results. In 1987, an on-the-spot jaw stretcher was made by bending two aluminium strips as shown and joining them by a hinge, so that an elastic band applied at one end led to the opening at the other end creating gradual pressure [Figures 16 and 18]. Figures 17 – 21, shows the pre operative views, post operative jaw stretching and the final views of the mouth opening.

In conclusion, inferiorly based temporalis fascia soft tissue interposition arthroplasty for temporomandibular joint ankylosis can be done under local anaesthesia and seems to be well accepted by the patients. Local anaesthesia for temporomandibular joint arthroplasty is a safe and simple procedure and avoids all the risks of general anaesthesia associated with difficult intubation.
